# Supplementary material for: Discovery of a Natural Syk Inhibitor from Chinese Medicine through a Docking-Based Virtual Screening and Biological Assay Study
Source: Molecules. 2018 Nov 28;23(12):3114. doi: 10.3390/molecules23123114 (PMC6320911; doi:10.3390/molecules23123114)
Supplement: Supplementary file 1 [file molecules-23-03114-s001.pdf]

Supplementary Materials

Discovery of a Natural Syk Inhibitor from Chinese Medicine through a Docking-based Virtual Screening and Biological Assay Study

Xing Wang [1,2], Junfang Guo [1], Zhongqi Ning [1] and Xia Wu [1,2,*]

[1] School of Traditional Chinese Medicine, Capital Medical University, Fengtai District, Beijing 100069, China; kingstar1016@sina.com (X.Wang); fanggj1519@ccmu.edu.cn (J.F.G.); ningzhongqi@163.com (Z.Q.N); wuxia6710@163.com (X.Wu)

[2] Beijing Key Lab of Traditional Chinese Medicine Collateral Disease Theory Research, Capital Medical University, Fengtai District, Beijing 100069, China

* Correspondence: wuxia6710@163.com (X.W.); Tel.: +86 10 83911633; Fax: +86 10 83911627

Table of Contents

S1. Fingerprint similarity search S1

S2. 3D pharmacophore model-based screening S2

S3. References S4

S1. Fingerprint similarity search

In order to verify if tanshinone I could be easily identified by applying fingerprint similarity search, the compounds from our in-house Chinese medicine database were filtered by using the 'find similar molecules by fingerprints' protocol in the library analysis module of Discovery Studio 2.5 (Accelrys, Inc. San Diego, CA, USA). The MDL public keys are used with the Tanimoto similarity coefficient [1]. The co-crystallized CG9 ligand in the crystal structure of Syk (PDB ID: 4PUZ) was used as a reference ligand. As shown in Figure S1, tanshinone I was ranked in the top 84.46%, which mean a poor virtual screening for Syk inhibitors.

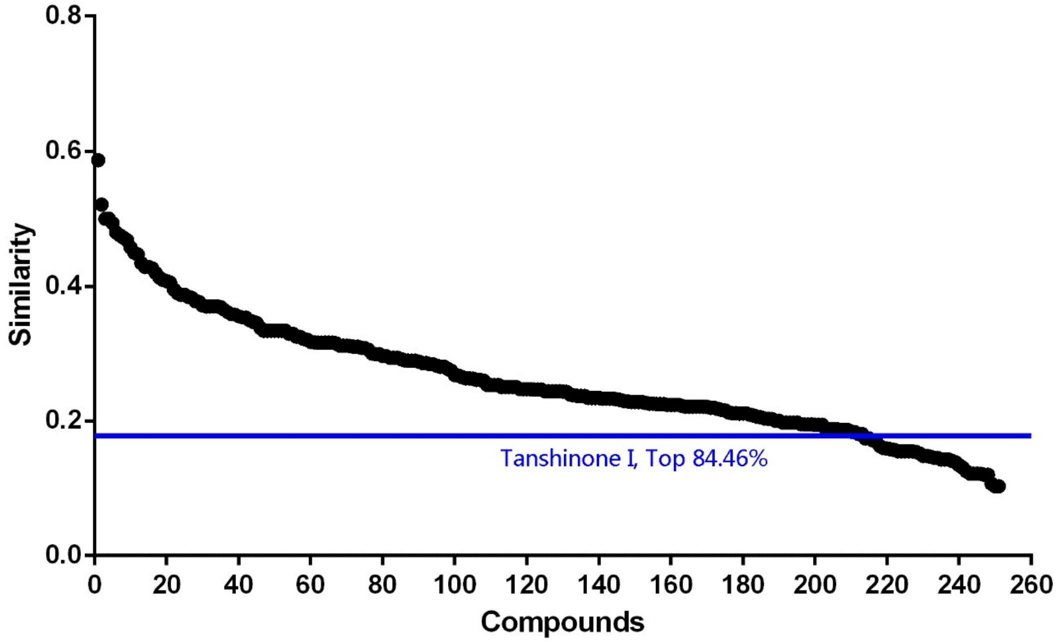

Figure S1. Fingerprint similarity search for Syk inhibitors.

S2. 3D pharmacophore model-based screening

In order to verify if tanshinone I could be easily identified by applying 3D pharmacophore model-based screening, a 3D pharmacophore model of Syk inhibitors was established by using the ‘Common Feature Pharmacophore Generation’ protocol in the pharmacophores module of Discovery Studio 2.5. Six experimentally known Syk inhibitors (Figure S2) were used as a training set to establish the 3D pharmacophore model[2-4]. The parameters were kept as the default settings.

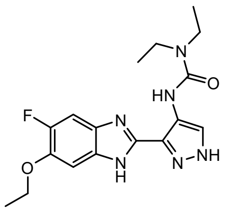

CHEMBL2017551
IC_{50}: 8 nM

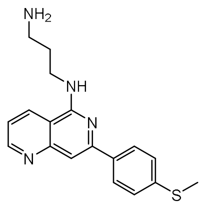

CHEMBL284590
IC_{50}: 34 nM

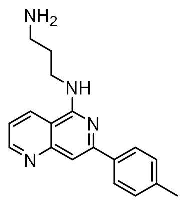

CHEMBL33466
IC_{50}: 63 nM

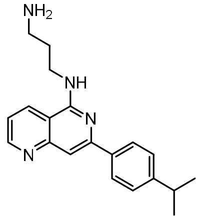

CHEMBL30873
IC_{50}: 90 nM

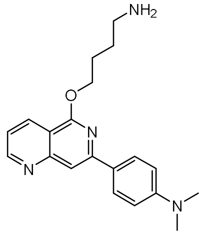

CHEMBL31141
IC_{50}: 160 nM

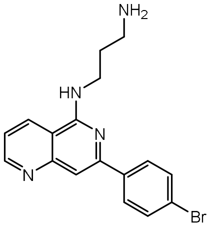

CHEMBL285346
IC_{50}: 260 nM

Figure S2. The training set used in pharmacophore model generation.

To validate the performance of the pharmacophore models established, an external decoy set database consisting of 292 experimentally known Syk inhibitors and 326 inactive compounds were screened utilizing the built-in parameters from our previous studies [5-16]. Four parameters (i.e. A%, Y%, N and CAI) were calculated to evaluate the generated models according to the following formula [17].

$$A\%=Ha/A\times100\%. \quad (1)$$

$$Y\%=Ha/Ht\times100\%. \quad (2)$$

$$N=(Ha\times D)/(Ht\times A). \quad (3)$$

$$CAI=N\times A\%. \quad (4)$$

D is the number of compounds in the external database. A is the number of active compounds. Ht is the number of hits and Ha is the number of active hits. A% represents the ability to identify active compounds from the external database. Y% represents the proportion of active hits in total hits. N represents the ability to distinguish active compounds from non-active compounds. CAI was utilized to evaluate the models comprehensively.

Ten pharmacophore models were generated based on the common features of six known Syk inhibitors. Model assessment studies (Table S1) indicated that Model_10 had the highest comprehensive appraisal index (CAI) and identified effective index (N), indicating that this model had the best ability to identify active compounds and exclude inactive compounds comprehensively. Model_10 was used to screen the compounds from our in-house Chinese medicine database, resulting in a hit list of 95 compounds (Figure S3) with tanshinone I ranked in the top 53.68%, which mean a poor virtual screening for Syk inhibitors.

Table S1. Assessment results for each pharmacophore model.

Model	Ht	Ha	A (%)	Y (%)	N	CAI
01	254	209	71.58	82.28	1.74	1.25
02	234	189	64.73	80.77	1.71	1.11
03	218	160	54.79	73.39	1.55	0.85
04	122	98	33.56	80.33	1.70	0.57
05	135	103	35.27	76.30	1.62	0.57
06	243	183	62.67	75.31	1.59	1.00
07	285	229	78.42	80.35	1.70	1.33
08	264	200	68.49	75.76	1.60	1.10
09	260	193	66.10	74.23	1.57	1.04
**10**	**330**	**262**	**89.73**	**79.39**	**1.68**	**1.51**

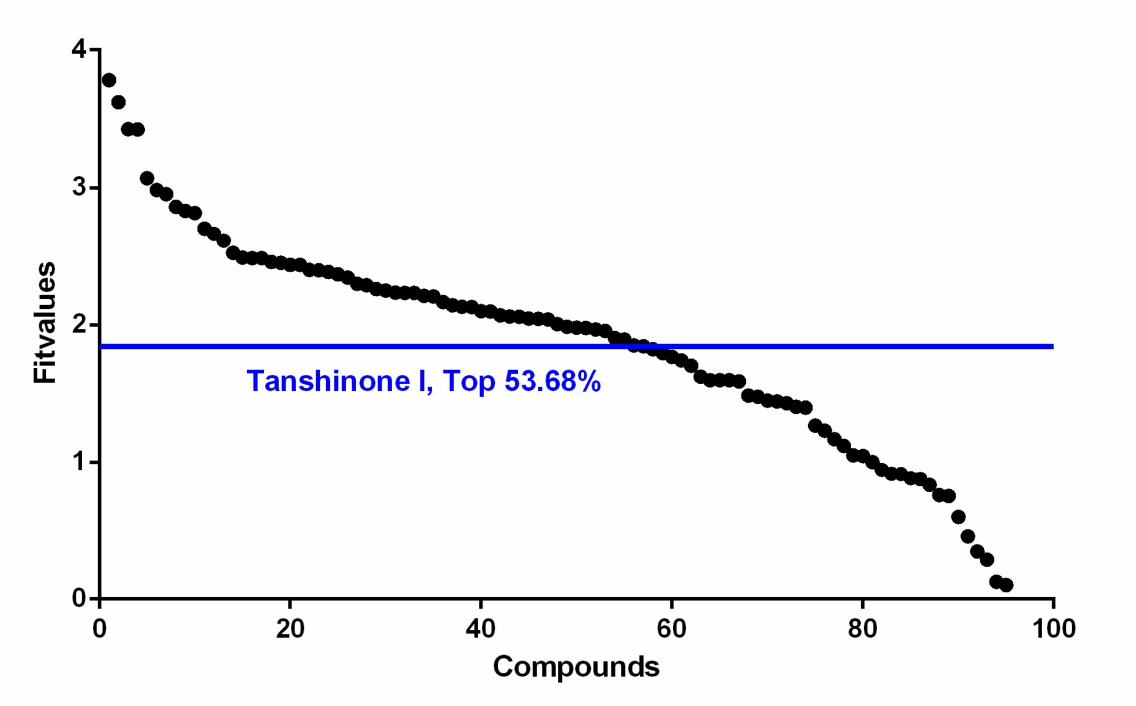

Figure S3. 3D pharmacophore model-based screening for Syk inhibitors.

S3. References

1. Paolini, G. V.; Shapland, R. H.; van Hoorn, W. P.; Mason, J. S.; Hopkins, A. L. Global mapping of pharmacological space. *Nat Biotechnol* **2006,** *24*, 805-15, http://dx.doi.org/10.1038/nbt1228.

2. Xie, H. Z.; Li, L. L.; Ren, J. X.; Zou, J.; Yang, L.; Wei, Y. Q.; Yang, S. Y. Pharmacophore modeling study based on known spleen tyrosine kinase inhibitors together with virtual screening for identifying novel inhibitors. *Bioorg Med Chem Lett* **2009,** *19*, 1944-9, http://dx.doi.org/10.1016/j.bmcl.2009.02.049.

3. Singh, R.; Masuda, E. S.; Payan, D. G. Discovery and development of spleen tyrosine kinase (SYK) inhibitors. *J Med Chem* **2012,** *55*, 3614-43, http://dx.doi.org/10.1021/jm201271b.

4. McLean, L. R.; Zhang, Y.; Zaidi, N.; Bi, X.; Wang, R.; Dharanipragada, R.; Jurcak, J. G.; Gillespy, T. A.; Zhao, Z.; Musick, K. Y.; Choi, Y. M.; Barrague, M.; Peppard, J.; Smicker, M.; Duguid, M.; Parkar, A.; Fordham, J.; Kominos, D. X-ray crystallographic structure-based design of selective thienopyrazole inhibitors for interleukin-2-inducible tyrosine kinase. *Bioorg Med Chem Lett* **2012,** *22*, 3296-300, http://dx.doi.org/10.1016/j.bmcl.2012.03.016.

5. Li, G. B.; Ji, S.; Yang, L. L.; Zhang, R. J.; Chen, K.; Zhong, L.; Ma, S.; Yang, S. Y. LEADOPT: an automatic tool for structure-based lead optimization, and its application in structural optimizations of VEGFR2 and SYK inhibitors. *Eur J Med Chem* **2015,** *93*, 523-38, http://dx.doi.org/10.1016/j.ejmech.2015.02.019.

6. Kaur, M.; Singh, M.; Silakari, O. Inhibitors of switch kinase 'spleen tyrosine kinase' in inflammation and immune-mediated disorders: a review. *Eur J Med Chem* **2013,** *67*, 434-46, http://dx.doi.org/10.1016/j.ejmech.2013.04.070.

7. Thoma, G.; Blanz, J.; Buhlmayer, P.; Druckes, P.; Kittelmann, M.; Smith, A. B.; van Eis, M.; Vangrevelinghe, E.; Zerwes, H. G.; Che, J. J.; He, X.; Jin, Y.; Lee, C. C.; Michellys, P. Y.; Uno, T.; Liu, H. Syk inhibitors with high potency in presence of blood. *Bioorg Med Chem Lett* **2014,** *24*, 2278-82, http://dx.doi.org/10.1016/j.bmcl.2014.03.075.
8. Liddle, J.; Atkinson, F. L.; Barker, M. D.; Carter, P. S.; Curtis, N. R.; Davis, R. P.; Douault, C.; Dickson, M. C.; Elwes, D.; Garton, N. S.; Gray, M.; Hayhow, T. G.; Hobbs, C. I.; Jones, E.; Leach, S.; Leavens, K.; Lewis, H. D.; McCleary, S.; Neu, M.; Patel, V. K.; Preston, A. G.; Ramirez-Molina, C.; Shipley, T. J.; Skone, P. A.; Smithers, N.; Somers, D. O.; Walker, A. L.; Watson, R. J.; Weingarten, G. G. Discovery of GSK143, a highly potent, selective and orally efficacious spleen tyrosine kinase inhibitor. *Bioorg Med Chem Lett* **2011,** *21*, 6188-94, http://dx.doi.org/10.1016/j.bmcl.2011.07.082.
9. Farmer, L. J.; Bemis, G.; Britt, S. D.; Cochran, J.; Connors, M.; Harrington, E. M.; Hoock, T.; Markland, W.; Nanthakumar, S.; Taslimi, P.; Ter Haar, E.; Wang, J.; Zhaveri, D.; Salituro, F. G. Discovery and SAR of novel 4-thiazolyl-2-phenylaminopyrimidines as potent inhibitors of spleen tyrosine kinase (SYK). *Bioorg Med Chem Lett* **2008,** *18*, 6231-5, http://dx.doi.org/10.1016/j.bmcl.2008.09.106.
10. Hirabayashi, A.; Mukaiyama, H.; Kobayashi, H.; Shiohara, H.; Nakayama, S.; Ozawa, M.; Tsuji, E.; Miyazawa, K.; Misawa, K.; Ohnota, H.; Isaji, M. Structure-activity relationship studies of imidazo[1,2-c]pyrimidine derivatives as potent and orally effective Syk family kinases inhibitors. *Bioorg Med Chem* **2008,** *16*, 9247-60, http://dx.doi.org/10.1016/j.bmc.2008.09.015.
11. Lucas, M. C.; Goldstein, D. M.; Hermann, J. C.; Kuglstatter, A.; Liu, W.; Luk, K. C.; Padilla, F.; Slade, M.; Villasenor, A. G.; Wanner, J.; Xie, W.; Zhang, X.; Liao, C. Rational design of highly selective spleen tyrosine kinase inhibitors. *J Med Chem* **2012,** *55*, 10414-23, http://dx.doi.org/10.1021/jm301367c.
12. Padilla, F.; Bhagirath, N.; Chen, S.; Chiao, E.; Goldstein, D. M.; Hermann, J. C.; Hsu, J.; Kennedy-Smith, J. J.; Kuglstatter, A.; Liao, C.; Liu, W.; Lowrie, L. E., Jr.; Luk, K. C.; Lynch, S. M.; Menke, J.; Niu, L.; Owens, T. D.; C, O. Y.; Railkar, A.; Schoenfeld, R. C.; Slade, M.; Steiner, S.; Tan, Y. C.; Villasenor, A. G.; Wang, C.; Wanner, J.; Xie, W.; Xu, D.; Zhang, X.; Zhou, M.; Lucas, M. C. Pyrrolopyrazines as selective spleen tyrosine kinase inhibitors. *J Med Chem* **2013,** *56*, 1677-92, http://dx.doi.org/10.1021/jm301720p.
13. Lucas, M. C.; Bhagirath, N.; Chiao, E.; Goldstein, D. M.; Hermann, J. C.; Hsu, P. Y.; Kirchner, S.; Kennedy-Smith, J. J.; Kuglstatter, A.; Lukacs, C.; Menke, J.; Niu, L.; Padilla, F.; Peng, Y.; Polonchuk, L.; Railkar, A.; Slade, M.; Soth, M.; Xu, D.; Yadava, P.; Yee, C.; Zhou, M.; Liao, C. Using ovality to predict nonmutagenic, orally efficacious pyridazine amides as cell specific spleen tyrosine kinase inhibitors. *J Med Chem* **2014,** *57*, 2683-91, http://dx.doi.org/10.1021/jm401982j.
14. Currie, K. S.; Kropf, J. E.; Lee, T.; Blomgren, P.; Xu, J.; Zhao, Z.; Gallion, S.; Whitney, J. A.; Maclin, D.; Lansdon, E. B.; Maciejewski, P.; Rossi, A. M.; Rong, H.; Macaluso, J.; Barbosa, J.; Di Paolo, J. A.; Mitchell, S. A. Discovery of GS-9973, a selective and orally efficacious inhibitor of spleen tyrosine kinase. *J Med Chem* **2014,** *57*, 3856-73, http://dx.doi.org/10.1021/jm500228a.
15. Ellis, J. M.; Altman, M. D.; Bass, A.; Butcher, J. W.; Byford, A. J.; Donofrio, A.; Galloway, S.; Haidle, A. M.; Jewell, J.; Kelly, N.; Leccese, E. K.; Lee, S.; Maddess, M.; Miller, J. R.; Moy, L. Y.; Osimboni, E.; Otte, R. D.; Reddy, M. V.; Spencer, K.; Sun, B.; Vincent, S. H.; Ward, G. J.; Woo, G. H.; Yang, C.; Houshyar, H.; Northrup, A. B. Overcoming mutagenicity and ion channel activity: optimization of selective spleen tyrosine kinase inhibitors. *J Med Chem* **2015,** *58*, 1929-39, http://dx.doi.org/10.1021/jm5018169.
16. Thoma, G.; Smith, A. B.; van Eis, M. J.; Vangrevelinghe, E.; Blanz, J.; Aichholz, R.; Littlewood-Evans, A.; Lee, C. C.; Liu, H.; Zerwes, H. G. Discovery and profiling of a

selective and efficacious Syk inhibitor. *J Med Chem* **2015,** *58*, 1950-63, http://dx.doi.org/10.1021/jm5018863.
17. Wang, X.; Zhang, Y. X.; Yang, Y.; Wu, X.; Fan, H. T.; Qiao, Y. J. Identification of berberine as a direct thrombin inhibitor from traditional Chinese medicine through structural, functional and binding studies. *Sci Rep-Uk* **2017,** *7*, http://dx.doi.org/10.1038/srep44040.
